# Knocking Down *Snrnp200* Initiates Demorphogenesis of Rod Photoreceptors in Zebrafish

**DOI:** 10.1155/2015/816329

**Published:** 2015-06-02

**Authors:** Yuan Liu, Xue Chen, Bing Qin, Kanxing Zhao, Qingshun Zhao, Jonathan P. Staley, Chen Zhao

**Affiliations:** ^1^Department of Ophthalmology, The First Affiliated Hospital of Nanjing Medical University and State Key Laboratory of Reproductive Medicine, Nanjing Medical University, Nanjing 210029, China; ^2^Department of Ophthalmology, The First People's Hospital of Suqian, Suqian 223000, China; ^3^Department of Ophthalmology, Tianjin Medical University, Tianjin Eye Hospital, Tianjin Key Laboratory of Ophthalmology and Visual Science, Tianjin 300040, China; ^4^Model Animal Research Center, MOE Key Laboratory of Model Animal for Disease Study, Nanjing University, Nanjing 210061, China; ^5^Department of Molecular Genetics and Cell Biology, University of Chicago, 920 E. 58th Street, Chicago, IL 60637, USA

## Abstract

*Purpose*. The small nuclear ribonucleoprotein 200 kDa (*SNRNP200*) gene is a fundamental component for precursor message RNA (pre-mRNA) splicing and has been implicated in the etiology of autosomal dominant retinitis pigmentosa (adRP). This study aims to determine the consequences of knocking down *Snrnp200* in zebrafish. *Methods*. Expression of the *Snrnp200* transcript in zebrafish was determined via whole mount *in situ* hybridization. Morpholino oligonucleotide (MO) aiming to knock down the expression of *Snrnp200* was injected into zebrafish embryos, followed by analyses of aberrant splicing and expression of the U4/U6-U5 tri-small nuclear ribonucleoproteins (snRNPs) components and retina-specific transcripts. Systemic changes and retinal phenotypes were further characterized by histological study and immunofluorescence staining. *Results*. *Snrnp200* was ubiquitously expressed in zebrafish. Knocking down *Snrnp200* in zebrafish triggered aberrant splicing of the *cbln1* gene, upregulation of other U4/U6-U5 tri-snRNP components, and downregulation of a panel of retina-specific transcripts. Systemic defects were found correlated with knockdown of *Snrnp200* in zebrafish. Only demorphogenesis of rod photoreceptors was detected in the initial stage, mimicking the disease characteristics of RP. *Conclusions*. We conclude that knocking down *Snrnp200* in zebrafish could alter regular splicing and expression of a panel of genes, which may eventually trigger rod defects.

## 1. Introduction

Retinitis pigmentosa (RP (MIM 268000)) is the most common form of inherited retinal dystrophies (IRDs) with a prevalence ranging from 1/3500 to 1/5000 among different populations [1–3]. RP presents significant clinical and genetic heterogeneities [4]. In the disease course of RP, rod photoreceptors will initially be affected followed by degeneration of cone photoreceptors and retinal pigment epithelium (RPE). The clinical hallmarks of RP include initial symptom of nyctalopia, subsequent constricted visual fields (VFs), and eventual loss of central vision. Hitherto, mutations in 64 genes have been found as RP causative (RetNet). Of those, 24 were autosomal dominant RP (adRP) relevant genes and they include seven ubiquitously expressed precursor messenger RNA (pre-mRNA) splicing genes, namely,* PRPF8* (MIM 607300) [5],* PRPF31* (MIM 606419) [6],* PRPF3* (MIM 607331) [7], PIM1-associated protein (*RP9* (MIM 607331)) [8], small nuclear ribonucleoprotein 200 kDa (*SNRNP200* (MIM 601664)) [9, 10],* PRPF6* (MIM 613979) [11], and* PRPF4* (MIM 607795) [12].

Recent genetic and functional studies have revealed the important role of pre-mRNA splicing in RP etiology and has shed light on the splicing process itself, a fundamental biological process [9, 12–14]. Pre-mRNA splicing is predominantly regulated by the spliceosome, a large complex that recognizes the pre-mRNA splice sites, removes the introns, and ligates the flanking exons accurately [15]. Notably, six of the seven identified adRP causative pre-mRNA splicing genes encode proteins embodied in the U4/U6-U5 tri-small nuclear ribonucleoproteins (snRNPs), a dynamic entity critical for the assembly and catalytic activation of the spliceosome, suggesting the important role of U4/U6-U5 tri-snRNP defects in the etiology and pathogenesis of RP [13]. The six genes can be further divided into two groups: U4/U6-specific genes including* PRPF3*,* PRPF4*, and* PRPF31* and U5-specific genes including* PRPF6*,* PRPF8*, and* SNRNP200*. How RP mutations in these ubiquitously expressed genes lead to retinopathy is currently under debate [12].

*SNRNP200*, located on 2q11.2 (RP33 locus), encodes the RNA helicase hBrr2, a U5-specific protein that contains 2136 amino acids and catalyzes the U4/U6 unwinding [16]. We have previously established that* SNRNP200* mutations do not compromise triple snRNP assembly but do compromise U4/U6 unwinding [9]. However, the retinal phenotypes and mRNA metabolism induced by knocking down* SNRNP200* in animal models have never been characterized. Therefore, to reveal the potential link between knockdown of* SNRNP200* and its relevant consequences, we studied the biochemical and morphological changes caused by knockdown of* Snrnp200* in a zebrafish model.

## 2. Materials and Methods

### 2.1. Whole Mount* In Situ* Hybridization in Zebrafish

All animal experiments were conducted in accordance with the Institutional Animal Care and Use Committee (IACUC) approved protocol and were approved and reviewed by the Institutional Committee of Nanjing Medical University. Zebrafish rearing and husbandry were maintained at 28.5°C with a 14-hour (hr) light/10 hr dark cycle. Digoxigenin-labeled antisense RNA probes were generated with cDNA of* Snrnp200* (NM_001123257.1), analogous of human* Snrnp200* in zebrafish, per the manufacturer's directions (Roche Applied Science, Mannheim, Germany). Whole mount* in situ* hybridization was conducted on 6 zebrafish for each developmental stage using a previously described modified protocol [17, 18]. Primer information is detailed in Supplementary Table S1 (in Supplementary Material available online at http://dx.doi.org/10.1155/2015/816329).

### 2.2. Morpholino Oligos and Knockdown of* Snrnp200* in Zebrafish

Standard control morpholino oligos (control-MO: 5′-CCTCTTACCTCAGTTACAATTTATA-3′) and zebrafish* Snrnp200* splicing-blocking MO targeting exon 25 (*Snrnp200*-MO: 5′-TCAACATCAAGACAACTCACATCCT-3′) were purchased from Gene Tools (Philomath, OR, USA). MO has been widely used to interfere in the transcription or translation of a targeted gene resulting in loss-of-function of the gene [19, 20]. Zebrafish embryos were injected at the 1- or 2-cell stage (0 day postfertilization (dpf)) with ~1 nL of purified MOs dissolved in water. To get the best adjusted dosage for* Snrnp200*-MO, zebrafish embryos were divided into three groups and injected with 2 (*n* = 67), 4 (*n* = 68), and 8 ng (*n* = 65) of MOs, respectively. Another two groups were further obtained and were injected with control-MO (4 ng; *n* = 72) and* Snrnp200*-MO (4 ng; *n* = 68), respectively. The counting and the percentage calculation of deformation and death in each injected group were conducted from 2 to 4 dpf as described previously [12]. Embryos that died within 24 hr postfertilization were excluded because such death likely resulted from unspecific causes. At 4 dpf, embryos with relatively normal appearance were collected from each injected group for further investigations.

### 2.3. RT-PCR and Q-PCR

RNA was isolated from 10 zebrafish embryos from each injection group, followed by reverse-transcriptase polymerase chain reaction (RT-PCR) to generate cDNA templates [9, 21]. PCR was subsequently conducted on the obtained cDNA to verify the effectiveness of* Snrnp200*-MO and amplify the targeted region of the* cbln1* gene with primers detailed in Supplementary Table S1 using a previously defined protocol [9, 21]. Quantitative real-time PCR (Q-PCR) was further performed using FastStart Universal SYBR Green Master (ROX; Roche, Basel, Switzerland) with the StepOnePlus Real-Time PCR System (Applied Biosystems, Darmstadt, Germany) per manufacturer's protocol. Expression analyses were conducted as described previously [22]. Primer information for Q-PCR was detailed in Supplementary Table S1.

### 2.4. Immunofluorescence Staining and Antibodies

Twelve embryos from the uninjected group, 11 from the control-MO injected group, and 14 from the* Snrnp200*-MO injected group were harvested and cryopreserved per standard procedures. They were fixed in 4% paraformaldehyde, incubated with 30% sucrose, embedded with optimal cutting temperature solution, and frozen in liquid nitrogen for sectioning at 5 *μ*m using a Leica CM1900 cryostat (Leica, Wetzlar, Germany). Rod and cone photoreceptors were further visualized through immunofluorescence staining as indicated previously [12]. Briefly, cryosections were incubated with designated primary antibodies, including antirhodopsin (Mouse, 1 : 250; Abcam, Cambridge, UK) and zpr-1 antibodies (Mouse, 1 : 250; ZRIC, USA), to label rod and cone photoreceptors, respectively. The cryosections were then treated with fluorescence-conjugated secondary antibodies (Invitrogen, Carlsbad, CA, USA) for another 1 hr at room temperature and finally counterstained by 4′,6-diamidino-2-phenylindole (DAPI; Sigma, USA) for cell nuclei staining. Images were taken with an Olympus IX70 confocal laser-scanning microscope (Olympus, Tokyo, Japan).

### 2.5. Statistics

GraphPad Prism (version 4.0; GraphPad Software, San Diego, CA, USA) was applied for statistical analysis. We also use one-way ANOVA or Student's *t*-test for comparisons among different groups. Data was presented as mean ± standard deviation (SD), and *P* < 0.05 was taken as statistically significant.

## 3. Results

### 3.1. Ubiquitous Expression of* Snrnp200* in Zebrafish

**Figure 1:**
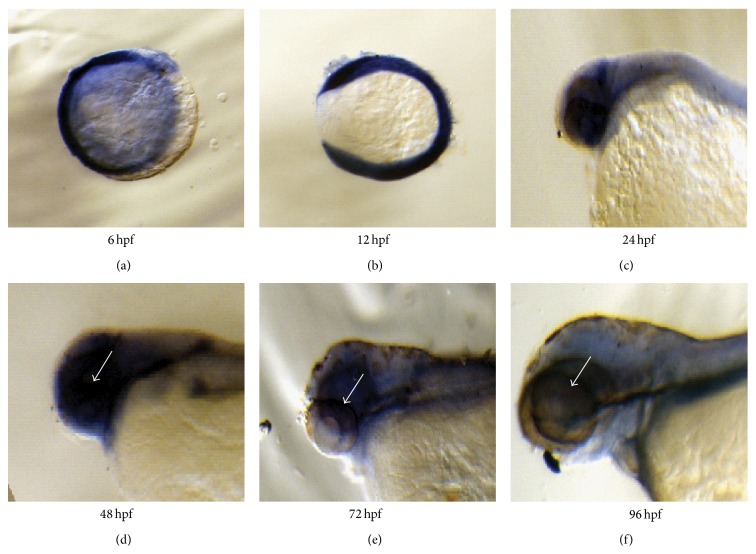
Whole mount* in situ* hybridization revealed a ubiquitous expression of the* Snrnp200* gene in all developmental stages of zebrafish. The 6 time points include 6 hours postfertilization (hpf) (a), 12 hpf (b), 24 hpf (c), 48 hpf (d), 72 hpf (e), and 96 hpf (f). Six embryos from each time point were collected for the experiment, and the* Snrnp200* signal in retina was indicated by white arrows (d–f).

We have previously showed a ubiquitous expression of* SNRNP200* in multiple human and murine tissues [9]. Whole mount* in situ* hybridization revealed overall expression of* Snrnp200* in zebrafish among all developmental stages (Figure 1).

### 3.2. Knocking Down* Snrnp200* Induces Aberrant Splicing in* cbln1*

**Figure 2:**
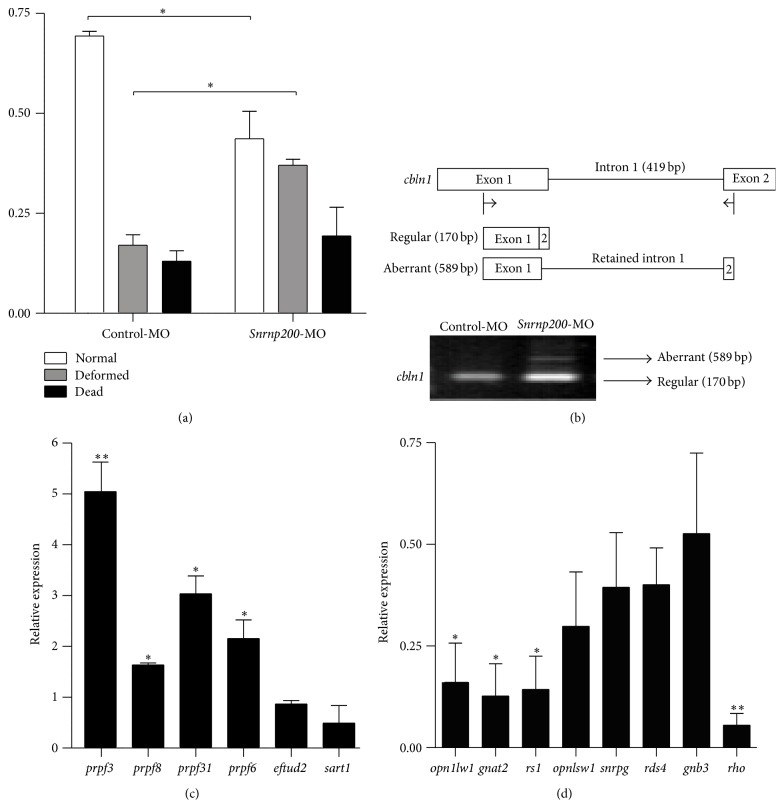
(a) Quantification of normal, deformed, and dead zebrafish injected with control morpholinos (control-MO) (*n* = 72) or* Snrnp200*-MO (*n* = 68) from 2 to 4 days postfertilization (dpf). (b) The upper panel presents the regular and aberrant cDNA structures of the* cbln1* gene, while the below panel indicates the aberrant splicing detected in the* cbln1* gene in zebrafish injected with* Snrnp200*-MO. The first intron of the* cbln1* gene was retained. (c) The relative expressions of splicing components, including* prpf3* (*P* = 0.007),* prpf8* (*P* = 0.011),* prpf31* (*P* = 0.032),* prpf6* (*P* = 0.048),* eftud2* (*P* = 0.086), and* sartl* (*P* = 0.125), were upregulated in zebrafish injected with* Snrnp200*-MO when compared with those injected with control-MO. (d) The relative expressions of retina specific transcripts, including* opn1lw1* (*P* = 0.013),* gnat2* (*P* = 0.011),* rs1* (*P* = 0.011),* opnlswl* (*P* = 0.060),* snrpg* (*P* = 0.068),* rds4* (*P* = 0.056),* gnb3* (*P* = 0.158), and* rho* (*P* = 0.002), were downregulated in zebrafish injected with* Snrnp200*-MO when compared with those injected with control-MO. Data in (a), (c), and (d) are presented as mean ± standard deviation (SD) for technical triplicates. ^*^ *P* < 0.05; ^**^ *P* < 0.01.

**Figure 3:**
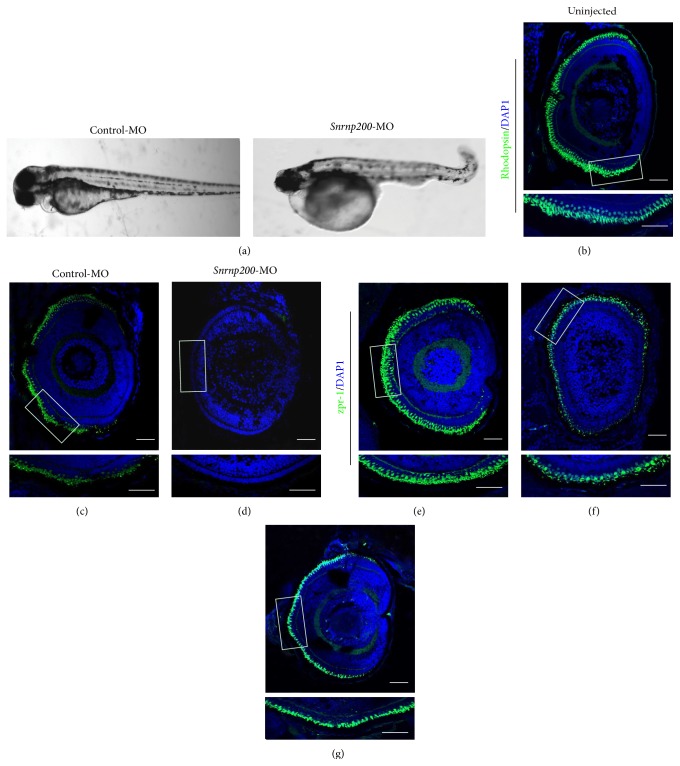
(a) Morphological changes in zebrafish injected with control morpholinos (control-MO) (left) or* Snrnp200*-MO (right) at 4 days postfertilization (dpf).* Snrnp200*-MO injection would result in morphological changes like malformed brains, short trunks, cardiac edema, and curved body axis, comparing with control-MO injection. (b–g) Retinal frozen sections of uninjected zebrafish (b and e) (*n* = 12) and zebrafish injected with control MO (c and f) (*n* = 11) and with* Snrnp200*-MO (d and g) (*n* = 14) were immunostained for rhodopsin (b–d) or zpr-1 (e–g).

The efficacy of* Snrnp200*-MO was confirmed by RT-PCR (Figure S1A) and the optimized condition for tissue-specific effects by* Snrnp200*-MO injection was defined with graded levels of concentration (Figures S1B and S1C). Injection of* Snrnp200*-MO at 4 ng in zebrafish was proved as the optimization dosage for an effective knockdown of the* Snrnp200* gene with moderate mortality (18%) and aberration (41.5%) rates (Figures 2(a), 3(a), and S1C). Previous studies demonstrated that patients with mutations in* PRPF31*,* PRPF3*,* PRPF8*, and* PRPF6* failed to correctly remove the intron between the first and the second exons of the* cbln1* gene [11]. Herein, the aberrantly spliced product (589 bp) containing the first intron of the* cbln1* gene was also detected in zebrafish injected with* Snrnp200*-MO (Figure 2(b)).

### 3.3. Knocking Down* Snrnp200* Triggers Upregulation of U4/U6-U5 Tri-snRNP Components and Downregulation of Retinal Transcripts

Knocking down a pre-mRNA splicing gene, like* sart3*,* prpf31*, or* prpf4*, in zebrafish can trigger compensatory responses by upregulating itself and other relevant splice components [12, 14, 23]. To test whether knocking down* Snrnp200* will have similar effects, we performed Q-PCR on the cDNA templates obtained from zebrafish injected with control-MO or* Snrnp200*-MO, respectively. Increased expressions of* prpf3* (NM_205748.1),* prpf31* (NM_200504.1),* prpf6* (NM_212655.1), and* prpf8* (NM_200976.2) were found in embryos injected with* Snrnp200*-MO when compared with those injected with control-MO, suggesting that other splicing components will show compensatory responses to* Snrnp200* deficiency in zebrafish (Figure 2(c)). In addition, consistent with previous findings in zebrafish with* prpf31* and* prpf4* knocked down [12, 14], Q-PCR revealed that the expression levels of several important retinal transcripts including* opn1lw1* (NM_131175.1),* gnat2* (NM_131869.2),* rs1* (NM_001003438.2), and* rho* (NM_131084.1) were decreased in* Snrnp200* morphants when compared with the control group (Figure 2(d)).

### 3.4. Demorphogenesis of Rod Photoreceptors in* Snrnp200* Morphants

We further analyzed whether knocking down* Snrnp200* in zebrafish will lead to primary damages on retina, especially defects in rod and cone photoreceptors. Retinal phenotypes were investigated on zebrafish injected with control-MO or* Snrnp200*-MO at 4 dpf. To confirm the integrity of the eyeballs, thus minimizing the possibility for false positive results, we only include larvae with relatively normal systemic appearances from each injected group to visualize the morphology of cone and rod photoreceptors. Larvae with severely general morphological defects, including malformed brains, short trunks, cardiac edema, and curved body axis, were excluded. The rhodopsin activity was significantly reduced in* Snrnp200*-morphants (Figures 3(b)–3(d)), while no significant divergence was shown concerning the morphology of the cone photoreceptors between the control and the morphant eyes (Figures 3(e)–3(g)).

## 4. Discussion

Mutations in the ubiquitously expressed pre-mRNA splicing genes have long been implicated in the etiology of RP, while the specific mechanisms underlying how such mutations would cause retina-specific phenotypes have not been fully elucidated. Previous studies suggest that reduced expression levels of* prpf31* and* prpf4* in zebrafish would selectively affect gene expression, in particular, retina-specific genes [12, 14]. However, both* prpf31* and* prpf4* are U4/U6-specific genes, and whether knocking down U5-specific genes will have similar impacts is still unknown. In the present study, we for the first time used a zebrafish model to characterize the retinal phenotypes and mRNA metabolism induced by knockdown of* Snrnp200*. Our findings in zebrafish indicate that knocking down of* Snrnp200* would cause systemic defects, retinal phenotypes, and splicing anomalies.

Similar to findings in zebrafish with* sart3*,* prpf31*, or* prpf4* knocked down [12, 14, 23], upregulation of other U4/U6-U5 tri-snRNP components was detected in* Snrnp200* morphants suggesting compensatory responses of other splicing components. Retinal phenotypes in the* Snrnp200* morphants were also similar to previous findings in* prpf31* and* prpf4* morphants [12, 14]. The expression levels of retina-specific transcripts were significantly reduced, and rod loss/demorphogenesis was predominantly detected in* Snrnp200* morphants, mimicking the RP phenotypes in patients. In addition, consistent with previous finding in patients carrying mutations in* PRPF31*,* PRPF3*,* PRPF8*, and* PRPF6*, aberrant splicing of the* cbln1* gene with retaining of its first intron was revealed in zebrafish injected with* Snrnp200*-MO [11]. Our result implies that defects in* SNRNP200* and other pre-mRNA splicing genes would share common splicing abnormalities.

The hBrr2 protein is critical for the proofreading of pre-mRNA splicing [9]. Thus,* SNRNP200* defects may interfere with the fidelity of retinal transcripts and further generate aberrantly spliced products toxic to the retina. Therefore, transcriptome analyses are needed for the detection of downstream target genes, particularly, retina-specific genes, of* SNRNP200* mutations and mutations in other adRP-associated splicing genes. Identification of such target genes will definitely provide us with a better insight into the molecular mechanisms for pre-mRNA splicing defects and could lead to discovery of new pathways for RP, which would assist in genetic counseling and direct future gene therapy for the patients with pre-mRNA splicing deficiency.

Taken together, our data suggest the potential pathogenesis shared by defects in pre-mRNA splicing genes, in particular, defects in* SNRNP200*,* PRPF31*, and* PRPF4*. Mutations in* PRPF31* have been reported to induce RP through haploinsufficiency [12], while* PRPF4* mutations are found as RP causative in a dominant negative manner [12]. Therefore, we highly hypothesize that* SNRNP200* mutations will cause RP eventually via loss-of-function, and investigations on the definite pathogenesis underlying* SNRNP200* mutations will be part of our future work. Elucidating the pathogenesis for* SNRNP200* mutations would aid in the development of novel therapeutics for retinitis pigmentosa. If haploinsufficiency mechanism is responsible for* SNRNP200* defects, the primary aim should be restoration of the normal levels of wild-type hBrr2 proteins. On the other hand, if the dominant negative gain-of-function toxicity mechanism plays an important role in photoreceptor loss, then enhancing the clearance of the toxic mutant hBrr2 protein would be of therapeutic value if implemented early.

## Supplementary Material

Morpholino Oligos and Knockdown of Snrnp200 Standard control morpholino oligos (control-MO: 5'-CCTCTTACCTCAGTTACAATTTATA-3') and zebrafish Snrnp200 splicing-blocking MO targeting exon 25 (Snrnp200-MO: 5'-TCAACATCAAGACAACTCACATCCT-3') were purchased from Gene Tools (Philomath, OR, USA). Zebrafish embryos were injected 3 at the 1- or 2-cell stage (0 day postfertilization (dpf)) with ∼ 1 nL of purified with ∼ 1 nl of purified MOs dissolved in water. To get the best adjusted dosage for Snrnp200-MO, zebrafish embryos were divided into three groups, and injected with 2 (n=67), 4 (n=68), and 8 ng (n=65) of MOs, respectively. The counting and the percentage calculation of deformation and death in each injected group were conducted from 2 to 4 dpf as described previously. Embryos died within 24 hr post fertilization were excluded because such death was likely resulted from unspecific causes. At 4 dpf, embryos with relatively normal appearance were collected from each injected group for further investigations.
RT-PCR: RNA was isolated from 10 zebrafish embryos from each injection group, followed by reverse-transcriptase polymerase chain reaction (RT-PCR) to generate cDNA template. PCR was subsequently conducted on the obtained cDNA to verify the effectiveness of Snrnp200-MO.
