# Supplementary material for: Knocking Down Snrnp200 Initiates Demorphogenesis of Rod Photoreceptors in Zebrafish
Source: J Ophthalmol. 2015 Jun 2;2015:816329. doi: 10.1155/2015/816329 (PMC4469172; doi:10.1155/2015/816329)
Supplement: Supplementary file 1 — Morpholino Oligos and Knockdown of Snrnp200 Standard control morpholino oligos (control-MO: 5'-CCTCTTACCTCAGTTACAATTTATA-3') and zebrafish Snrnp200 splicing-blocking MO targeting exon 25 (Snrnp200-MO: 5'-TCAACATCAAGACAACTCACATCCT-3') were purchased from Gene Tools (Philomath, OR, USA). Zebrafish embryos were injected 3 at the 1- or 2-cell stage (0 day postfertilization (dpf)) with ∼ 1 nL of purified with ∼ 1 nl of purified MOs dissolved in water. To get the best adjusted dosage for Snrnp200-MO, zebrafish embryos were divided into three groups, and injected with 2 (n=67), 4 (n=68), and 8 ng (n=65) of MOs, respectively. The counting and the percentage calculation of deformation and death in each injected group were conducted from 2 to 4 dpf as described previously. Embryos died within 24 hr post fertilization were excluded because such death was likely resulted from unspecific causes. At 4 dpf, embryos with relatively normal appearance were collected from each injected group for further investigations. RT-PCR: RNA was isolated from 10 zebrafish embryos from each injection group, followed by reverse-transcriptase polymerase chain reaction (RT-PCR) to generate cDNA template. PCR was subsequently conducted on the obtained cDNA to verify the effectiveness of Snrnp200-MO. [file 816329.f1.pdf]

SI Figure, Legends and Tables

Legends to Supplementary Figure S1

FIGURE S1: A. Regular (upper panel) and aberrant spliced product (lower panel) generated by zebrafish injected with control morpholinos (MO) or *snrnp200* MO, respectively. B. Morphological changes in zebrafish injected with control morpholinos (MO; dosage: 4 ng) or *snrnp200* MO (dosages: 2, 4, and 8 ng, respectively) at 4 days post fertilization (dpf). C. Quantification of normal, deformed and dead zebrafish injected with control morpholinos (MO; dosage: 4 ng) or *snrnp200* MO (dosages: 2, 4, and 8 ng, respectively) from 2 to 4 days post fertilization (dpf). Data in C are presented as mean ± standard deviation (SD) from technical triplicates. $* P < 0.05$.

Supplementary Figure S1

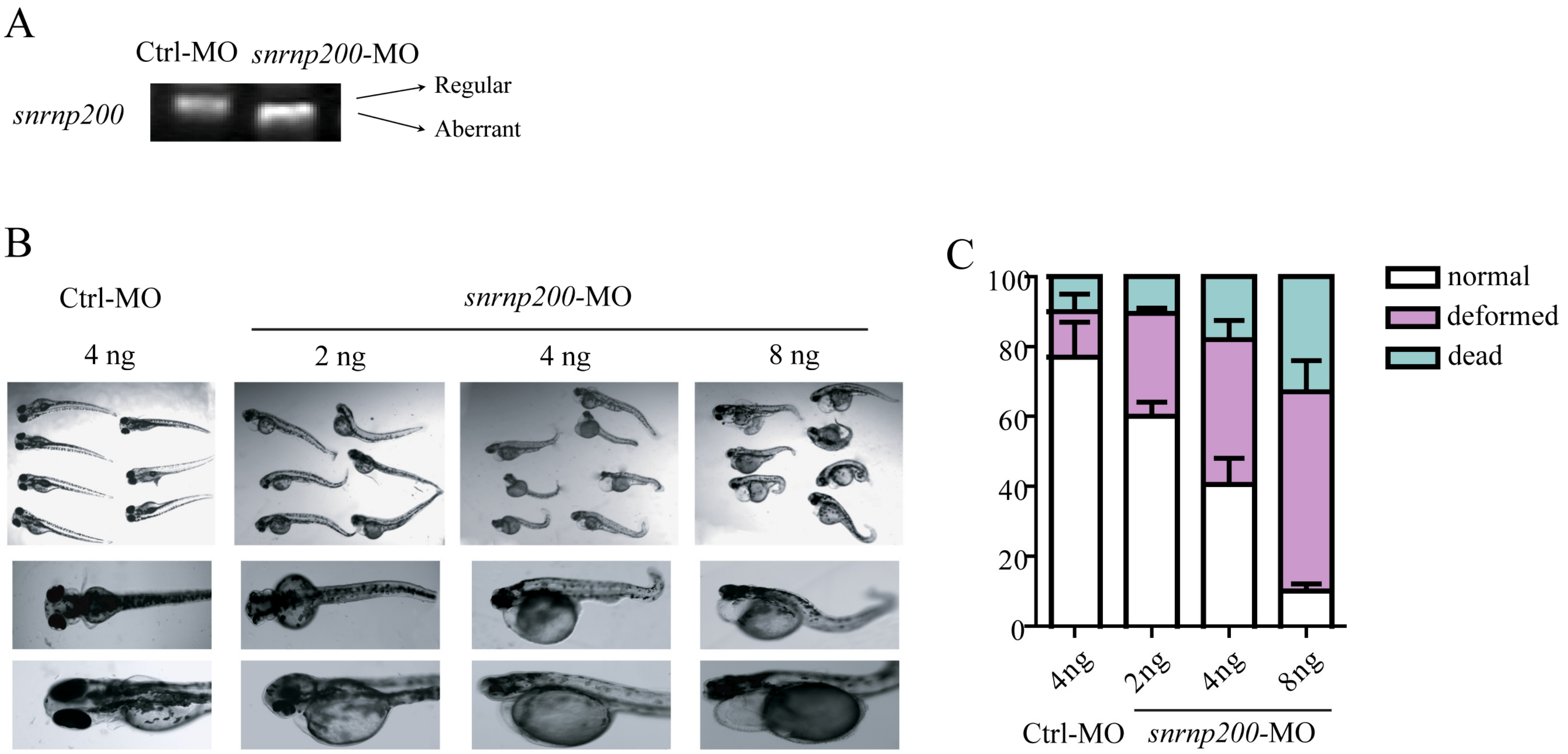

Supplementary TABLE S1: Summary of Primer Information.		
Gene / Plasmid	Forward Primer (5'→3')	Reverse Primer (5'→3')
For In Situ Probe Synthesis		
snrnp200	ACCGGGTGTGCTGGTTAATA	CGATGGCATCGTTGTAGAAG
For Verification of the Effectiveness of Morpholino Oligos		
snrnp200-MO	AGCGAACTCTAACTTGGT	TTCTCATCAGTCTCCATAGTGGTTT
For Verification of the Splicing Defects Caused		
cbln1-E1/E2	TCAAATCCCACCTCAGACCC	CTTCTACCAACGTTGACCAGT
For Q-PCR Assay		
β-actin	CGAGCTGTCTTCCCATCCA	TCACCAACGTAGCTGTCTTTCTG
prpf3	GCTAAGTTTTGCGGCTCCTA	TCCAGGGTTTCCCACAGTAG
prpf31	CAAGCAGGTCAAGCCTCTTC	TTGTCTGACTCTGCCACTGC
prpf6	GAGAAAGTTGGGCAGCTCAC	GACATTCCTGAAGGGCTTTG
prpf8	TTCCATGGGACACGTACTGA	CTGTCGCTTGAGGGCATATT

eftud2	CTACACGGGTGGACTGGACT	TTTCTCCCAGAACCTTCACG
sart1	AGAAGGTGGCTCGTGTCAAG	CCCCGATTCATCAACGTATT
opn1lw1	AAACCACAAGGGAAGCAATG	GGATCCAGTTGAGAGGGTGA
gnat2	AGTCTGCTCTGGCCATCATC	AGCAGAGTCGTTCAGCTGGT
rs1	CCTGAAGGAGGTGAAAGTGG	TGGAAGAGCGGTCAGAGTTC
opnlsw1	TTCCTTCTCGTCACCTGCTT	AATCCTTGTTTGGCTCATCG
snrpg	GAACAACGCTTGAAACGTGA	CATGAAGGGGTCAAATCCAC
rds4	ACCACCTGACCAACAACTCC	CAACCCTCACTTTCGCTCTC
gnb3	TGTCCTGTTGCCGTTTCATA	ACTTGTGTGACCCTGGAAGG
rho	CGTGCACTTCTTCATCCCAC	AGGAAGGCGATGACCATGAT
